# EBNA2 Binds to Genomic Intervals Associated with Multiple Sclerosis and Overlaps with Vitamin D Receptor Occupancy

**DOI:** 10.1371/journal.pone.0119605

**Published:** 2015-04-08

**Authors:** Vito A. G. Ricigliano, Adam E. Handel, Geir K. Sandve, Viviana Annibali, Giovanni Ristori, Rosella Mechelli, M. Zameel Cader, Marco Salvetti

**Affiliations:** 1 Nuffield Department of Clinical Neurosciences, University of Oxford, John Radcliffe Hospital, Oxford OX3 9DU, United Kingdom; 2 Neuroimmunology Unit, Fondazione Santa Lucia (I.R.C.C.S.), Rome, Italy; 3 Medical Research Council Functional Genomics Unit and Department of Physiology, Anatomy and Genetics, University of Oxford, Oxford OX1 3PT, United Kingdom; 4 Weatherall Institute of Molecular Medicine, University of Oxford, John Radcliffe Hospital, Oxford OX3 9DS, United Kingdom; 5 Department of Informatics, University of Oslo, Blindern, Norway; 6 Centre for Experimental Neurological Therapies (CENTERS), Neurology and Department of Neuroscience, Mental Health and Sensory Organs, Faculty of Medicine and Psychology, “Sapienza” University of Rome, Rome, Italy; Nihon University School of Medicine, JAPAN

## Abstract

Epstein-Barr virus (EBV) is a non-heritable factor that associates with multiple sclerosis (MS). However its causal relationship with the disease is still unclear. The virus establishes a complex co-existence with the host that includes regulatory influences on gene expression. Hence, if EBV contributes to the pathogenesis of MS it may do so by interacting with disease predisposing genes. To verify this hypothesis we evaluated EBV nuclear antigen 2 (EBNA2, a protein that recent works by our and other groups have implicated in disease development) binding inside MS associated genomic intervals. We found that EBNA2 binding occurs within MS susceptibility sites more than expected by chance (factor of observed *vs* expected overlap [O/E] = 5.392-fold, p < 2.0e-05). This remains significant after controlling for multiple genomic confounders. We then asked whether this observation is significant per se or should also be viewed in the context of other disease relevant gene-environment interactions, such as those attributable to vitamin D. We therefore verified the overlap between EBNA2 genomic occupancy and vitamin D receptor (VDR) binding sites. EBNA2 shows a striking overlap with VDR binding sites (O/E = 96.16-fold, p < 2.0e-05), even after controlling for the chromatin accessibility state of shared regions (p <0.001). Furthermore, MS susceptibility regions are preferentially targeted by both EBNA2 and VDR than by EBNA2 alone (enrichment difference = 1.722-fold, p = 0.0267). Taken together, these findings demonstrate that EBV participates in the gene-environment interactions that predispose to MS.

## Introduction

Epstein-Barr virus (EBV) is associated with the development of multiple sclerosis (MS), as supported by epidemiological surveys, serological evidences and other experimental laboratory based studies [1–6]. However, its exact contribution to the pathogenesis of MS remains unclear.

Investigating the possible interplay between EBV and genetic susceptibility factors identified by genome-wide association studies (GWAS) may help clarify its possible causative role. Recently, using a "candidate interactome" approach, our group has shown that the interaction between disease-associated genetic variants and EBV proteins is relevant for MS etiology [7]. Viral proteins are able to bind DNA by means of direct [8] or indirect [9] interactions, and localize to promoter and enhancer regions [10] in infected cells, thus affecting transcriptional programs [11].

Among EBV nuclear proteins, Epstein-Barr nuclear antigen 2 (EBNA2) plays a key role in B cell transformation. Furthermore, some evidences suggest its potential implication in MS pathology: EBNA2 expressing cells have been observed in affected brains [12] and specific EBNA2 genotypes associate with disease status [13]. However, so far no analyses have been performed to define whether EBNA2 preferentially controls the expression of genes associated with MS susceptibility.

To fill this gap, using publicly available ChIP-seq data of lymphoblastoid cell lines (LCLs), we characterized the genomic distribution of EBNA2—and of its cognate factor recombination signal binding protein for immunoglobulin kappa J region (RBPJ)[9]—within disease-specific regions. We then investigated its potential interplay with vitamin D, another major environmental risk factor for MS, whose receptor (VDR) preferentially interacts with disease susceptibility regions in LCLs [14] and other immune cell types [15]. To study their relation on a genomic scale, we looked at the overlap between EBNA2 distribution and VDR occupancy both inside and outside of MS associated regions.

## Methods

MS without major histocompatibility complex (MHC) single nucleotide polymorphisms (SNPs) were obtained from the latest published list of GWAS variants [16]; MS MHC SNPs included 2 Class II SNPs (rs3135388 and rs3135391) and 20 Class I SNPs contributing to MS risk independently of the HLA-DRB1*15:01 [17]. For the other disorders (obesity, UC, RA and SLE), we collected disease-associated SNPs from the ‘Catalog of Published Genome-Wide Association Studies’ (http://www.genome.gov/gwastudies/)[18]. Only associations reported from GWAS with replication cohort were selected. Associated regions were defined as the genomic intervals of 25, 50, 100 and 150 kb centered on the MS associated SNPs. Genomic interval of 100 kb were considered for other disease- associated SNPs. GM12878 DNase I hypersensitive sites (DHSs) track and immune response gene track were extracted from ENCODE data [19] and the HyperBrowser track repository [20].

EBNA2 and RBPJ ChIP-seq raw data were obtained from a previous publication [9] and processed on the Lifeportal platform (https://lifeportal.uio.no). We used BWA [21] for Illumina to map sequences to the reference genome hg19 and MACS (Model-based analysis of ChIP-Seq) [22] for peak-calling. MACS parameters were defined as follows: default genome size, 26 bp tag size, 300 bp band width and a p-value cutoff for peak detection of 1e-05. Peaks with a false discovery rate (FDR) of <1% were selected. VDR genomic distribution was retrieved from the literature [23]. All the analyses on processed ChIP-seq data and disease regions were performed using the Genomic HyperBrowser (http://hyperbrowser.uio.no/hb/)[20]. Enrichment between two different tracks was determined accordingly to what has been described elsewhere [14] and was expressed as the factor of observed *vs* expected overlap (O/E), which calculates the degree of coverage by track A inside track B segments *vs* outside them.

In order to assess statistical significance, we defined a null model where the location of track A intervals varied randomly, while preserving inter-segment gaps distribution and segment lengths. Track B intervals were kept fixed. Overlap analysis between the tracks was measured on real data, as well as on 50.000 Monte Carlo samples from the null model. Statistical significance was taken at p<0.05.

To estimate the overlap of ChIP-seq sites (EBNA2 or RBPJ) and MS associated regions relative to that measured for other disorder-associated regions or for immune response gene regions, we generated case–control tracks for each disease pair (MS track-non MS track) and for the MS-immune response gene pair by discarding the overlapping segments between tracks and marking the remaining intervals as case (MS specific intervals) or control (other disorder specific intervals, immune response gene specific intervals). We then calculated the fold enrichment difference for the overlap with ChIP-seq regions between MS associated intervals *vs* control intervals.

To control for any confounding effects of chromatin state (‘open’ *vs* ‘closed’) on EBNA2-MS, EBNA2-VDR and RBPJ-MS relations, we adjusted for confounding track GM12878 DHSs, using the strategy for confounder handling previously described [24]. In the statistical hypothesis testing, MS regions (or VDR segments, for the EBNA2-VDR relations) were kept fixed.

Plots of VDR, RBPJ and DHSs peak distribution around EBNA2 peak position were drawn using Homer (http://homer.salk.edu/homer/ngs/quantification.html).

Prediction of function of *cis*-regulatory regions for EBNA2, VDR and the joint overlap track EBNA2-VDR was performed using GREAT [25].

For binding motif analysis, we first enlarged the SNP position track (including both confirmed MS SNPs and those in LD [r^2^≥ 0.8] with them, obtained from SNAP [26] on CEU individuals) of 100 bp on each side, in order to intersect it with RBPJ ChIP-seq peaks. We then used MEME and FIMO tools (http://meme.nbcr.net/meme/intro.html) to screen for potential disruptive effects of MS variants on the RBPJ motif.

## Results

We performed an overlap analysis of EBNA2 and RBPJ ChIP-seq peaks and found a 85.6% EBNA2-RBPJ joint occupancy at the base pair (bp) level, calculated on the overall number of bp covered by EBNA2 peaks (S1A Table). This result confirmed literature data [9], thus indicating the reliability of our method. We also measured the enrichment (i.e., degree of coverage by a track within the segments of a second track *vs* outside them) of EBNA2 peaks inside RBPJ binding sites and demonstrated a high co-localization of EBNA2 and RBPJ (factor of observed *vs* expected overlap [O/E] = 431.4, p < 2.0e-05, S1B Table).

Specifically looking at the distribution of EBNA2 and RBPJ in relation to MS associated regions, defined as described in Methods, we performed an enrichment analysis of their binding sites inside disease associated intervals, using two different MS tracks, one including and the other one excluding the MHC class I and class II regions. This distinction was made in order to account for any predominant effects of MHC single nucleotide polymorphisms (SNPs), which show the strongest association with the disease. We found that MS susceptibility loci are substantially enriched for both EBNA2 (O/E calculated for MS regions, MHC removed = 5.392-fold, p < 2.0e-05; MHC included = 5.047-fold, p < 2.0e-05) and RBPJ (O/E calculated for MS regions, MHC removed = 6.841-fold, p < 2.0e-05; MHC included = 6.402-fold, p < 2.0e-05) (Table 1).

**Table 1:** EBNA2/RBPJ overlap with MS regions.

ANALYSIS DESCRIPTION	enrichment (O/E)	*p*-value	correction for confounder track GM12878 DHSs
‘RBPJ peaks' inside 'MS non-MHC regions'	**6.841**	**2.00e-05**	**9.99e-04**
'EBNA2 peaks' inside 'MS non-MHC regions'	**5.392**	**2.00e-05**	**9.99e-04**
'RBPJ peaks' inside 'MS regions including MHC'	**6.402**	**2.00e-05**	**9.99e-04**
'EBNA2 peaks' inside 'MS regions including MHC'	**5.047**	**2.00e-05**	**9.99e-04**

Results of EBNA2/RBPJ peak overlap with MS associated regions, defined as SNP position ± 50kb

O/E: factor of observed *vs* expected overlap

EBNA2 = Epstein-Barr nuclear antigen 2; RBPJ = Recombination signal-binding protein for immunoglobulin Kappa J region; DHS = DNase I hypersensitive sites; MHC = Major histocompatibility complex; MS = multiple sclerosis.

To verify the sensitivity of our results with respect to a choice (SNP interval 100 kb) that is not obvious based on the literature published so far, we evaluated different SNP intervals (25, 50 and 150 kb). These analyses supported the consistency of the results (S2 Table), showing that SNP intervals do not affect the enrichment of EBNA2 and RPBJ within MS susceptibility loci.

Specific regions of intersection between EBNA2 or RBPJ peaks and MS regions/MS SNPs—both original GWAS SNPs or SNPs in linkage disequilibrium (LD)≥0.8 with them—are listed in detail in S3A–F Table.

We then assessed whether any of the SNPs intersecting RBPJ peaks induced a disruption of its binding motif, but we did not find any significant alterations (none of the SNPs showed a *q*-value <0.05, S4 Table).

A potential confounder for the EBNA2/RBPJ overlap with MS intervals is that transcription factors normally tend to bind to regions of open chromatin. To address this issue, we controlled for regions of open chromatin in LCLs, using data on DNase hypersensitivity peaks (GM12878 DHSs) from the ENCODE project [19]. This represents a reliable strategy to rule out gene proximity effects. Even when adjusting for DHSs track, the enrichment for both EBNA2 and RBPJ binding within MS susceptibility regions remained significant (p = 9.99e-04, Table 1). Another possible confounder for the results of EBNA2 overlap with MS associated sites could be the co-localization of viral proteins with immune-related genes. We indeed found an enrichment of EBNA2 in immune regions (O/E = 3.261-fold, *p* = 0.00012) (Table 2). To control for this, we normalized the EBNA2-enriched MS intervals for a general track of immune response gene regions derived from the Genomic HyperBrowser track repository [20]. We found an excess enrichment of EBNA2 binding to MS regions relatively to the immune gene track: enrichment difference (EED) = 1.603-fold p = 6.0e-05 (Table 2). This result shows that EBNA2 enrichment inside MS loci is not simplistically dependent on its tendency to interact with regions mapping to immune-related genes.

**Table 2:** EBNA2 peak overlap with immune response gene regions and excess enrichment of case-control analysis.

ANALYSIS DESCRIPTION	enrichment (O/E)	p-value
'EBNA2 peaks' inside 'immune response gene regions'	**3.261**	**0.00012**
Preferential overlap of 'EBNA2 peaks' on 'MS regions including MHC' (case) relatively to 'immune response gene regions' (control)	**1.603** ^**1**^	**6.00e-05**

O/E: factor of observed *vs* expected overlap; ^1^: enrichment difference (EED).

EBNA2 = Epstein-Barr nuclear antigen 2; RBPJ = Recombination signal-binding protein for immunoglobulin Kappa J region; MHC = Major histocompatibility complex; MS = multiple sclerosis.

To verify whether our findings could be MS-specific or common to other diseases sharing with MS a multifactorial etiology, we took into account four complex disorders, characterized by a different contribution of genetic, environmental and immune factors: obesity, ulcerative colitis (UC), rheumatoid arthritis (RA) and systemic lupus erythematosus (SLE). Specifically, RA and SLE share with MS part of the genetic background (i.e., MHC region) and the association with EBV as major environmental risk factor [27, 28]. On selected disorders, we measured EBNA2 overlap and found no significant enrichment inside obesity and UC susceptibility loci (O/E for obesity = 0.4565-fold, *p* = 0.8378; O/E for UC = 1.892-fold, *p* = 0.06522); on the contrary, RA and SLE regions showed results comparable to those seen in MS (O/E for RA, MHC included = 4.776-fold, *p* = 9.99e-05; O/E for SLE, MHC included = 6.4-fold, *p* = 9.99e-05) (Table 3); analyses performed without considering MHC regions yielded similar results (data not shown). We then performed case-control enrichment analyses using MS regions with MHC as cases and obesity, UC, RA and SLE as controls. We observed an excess enrichment of EBNA2 binding within MS associated regions relatively to control tracks only for obesity and UC (EED relative to obesity track = 10.98-fold p = 0,00028; EED relative to UC track = 2.888-fold, p = 0,03684; EED relative to RA track = 1.044-fold, p = 0.2174; EED relative to SLE track = 0.8339-fold, p = 0,3436) (Table 3). Our results show that EBNA2 preferentially binds to regions genetically associated with diseases sharing EBV as major environmental risk factor.

**Table 3:** EBNA2 overlap with genomic region associated to other disorders.

ANALYSIS DESCRIPTION	enrichment (O/E)	p-value	case/control analysis ^1^	p-value
'EBNA2 peaks' inside 'obesity egions'	**0.457**	**0.8378**	**10.98**	**0.00028**
'EBNA2 peaks' inside 'UC regions including MHC'	**1.887**	**0.06522**	**2.888**	**0.03684**
'EBNA2 peaks' inside 'RA regions including MHC'	**4.776**	**9.99e-05**	**1.044**	**0.2174**
'EBNA2 peaks' inside 'SLE regions including MHC'	**6.4**	**9.99e-05**	**0.8339**	**0.3436**

Results of the overlap analysis between EBNA2 peaks and other disorder-associated regions.

O/E: factor of observed *vs* expected overlap ^1^: excess enrichment inside MS regions (case) relatively to control regions.

EBNA2 = Epstein-Barr nuclear antigen 2; UC = ulcerative colitis; MHC = Major histocompatibility complex; RA = Rheumatoid arthritis; SLE = Systemic lupus erythematosus.

In the attempt to identify a more MS-specific signature in EBNA2 distribution, we then investigated a possible overlap with VDR genomic occupancy in the same cell types [23]. We first intersected EBNA2 and VDR ChIP-seq peaks and then measured their binding inside MS regions (performing the evaluation with or without the MHC region, as above): a significant enrichment (O/E without MHC = 8.667-fold, *p* = 8.88e-03; O/E with MHC = 8.112-fold, *p* = 8.64e-03) was found, even after controlling for DHSs track (Table 4). When performing a case-control analysis using EBNA2-VDR joint track as case and EBNA2 as control, we found an excess enrichment of the first track inside MS intervals relatively to EBNA2 single binding (EED on MS regions with MHC = 1.722-fold, *p* = 0.0267) (Table 4). We then evaluated EBNA2-VDR joint overlap with RA and SLE regions, given the evidence of a role for vitamin D in these disorders. Analyzed intervals showed no significant enrichment: O/E for RA without MHC = 1.29-fold, *p* = 0.3443; O/E for RA with MHC = 1.232-fold, *p* = 0.3426; O/E for SLE without MHC = 3.329-fold, *p* = 0.3849; O/E for SLE with MHC = 3.195-fold, *p* = 0.3776. We compared these results to those observed for MS, using the same case-control strategy described above, setting MS regions as case and RA or SLE as control. We found an excess enrichment of EBNA2-VDR joint overlap inside MS regions relatively to each control track: EED relative to RA track with MHC = 6.679-fold p = 0,0213; EED relative to SLE track with MHC = 2.321-fold, p = 0,1772 (not significant) (Table 5).

**Table 4:** EBNA2-VDR joint binding inside MS and other disease-associated regions.

ANALYSIS DESCRIPTION	enrichment (O/E)	*p*-value	correction for confounder track GM12878 DHSs	case/control analysis ^1^	p-value
'EBNA2 and VDR joint occupancy track' inside 'MS non-MHC regions'	**8.667**	**8.88e-03**	**9.99e-04**	**1.722**	**0.0267**
'EBNA2 and VDR joint occupancy track ' inside 'MS regions including MHC'	**8.112**	**8.64e-03**	**9.99e-04**	**1.722**	**0.0253**

Overlap results for EBNA2-VDR joint occupancy track within MS regions and excess enrichment relatively to EBNA2 exclusive peaks.

O/E: factor of observed *vs* expected overlap; ^1^: EED for EBNA2-VDR peaks (case) relatively to control (EBNA2 exclusive peaks) inside MS regions.

EBNA2 = Epstein-Barr nuclear antigen 2; VDR = Vitamin D receptor; MS = multiple sclerosis; DHS = DNase I hypersensitive sites

**Table 5:** EBNA2-VDR joint binding inside RA and SLE associated regions.

ANALYSIS DESCRIPTION	enrichment (O/E)	p-value	case/control analysis ^1^	p-value
'EBNA2 and VDR joint occupancy track' inside 'RA non MHC regions'	**1.29**	**0.3443**	**6.841**	**0.0193**
'EBNA2 and VDR joint occupancy track' inside 'RA regions including MHC'	**1.232**	**0.3426**	**6.679**	**0.0213**
'EBNA2 and VDR joint occupancy track' inside 'SLE non MHC regions'	**3.329**	**0.3849**	**2.366**	**0.1805**
'EBNA2 and VDR joint occupancy track' inside 'SLE regions including MHC'	**3.195**	**0.3776**	**2.321**	**0.1772**

Overlap results for EBNA2-VDR joint occupancy track within RA and SLE regions, and excess enrichment of case-control analysis

O/E: factor of observed *vs* expected overlap; ^1^: EED for EBNA2-VDR joint occupancy track on MS regions (case) relatively to control regions (RA, SLE).

EBNA2 = Epstein-Barr nuclear antigen 2; VDR = Vitamin D receptor; RA = Rheumatoid arthritis; SLE = Systemic lupus erythematosus; MHC = Major histocompatibility complex.

These findings suggest that EBNA2-VDR joint occupancy sites are more selectively located inside MS associated regions when compared to other EBV and vitamin D-related autoimmune diseases. Furthermore, they indicate that EBNA2 and VDR partially share the occupancy sites on the DNA.

To confirm this in a broad perspective, we intersected their distribution on the genomic scale.

**Fig 1:**
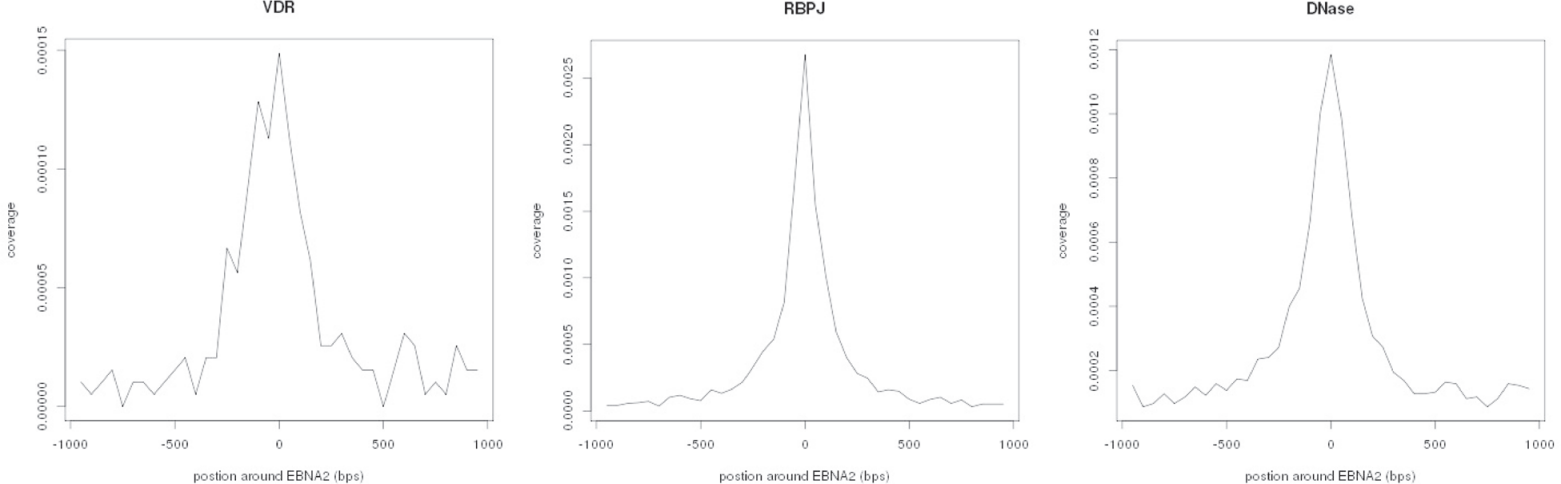
Histogram plots of VDR, RBPJ and DNase peak distribution centered on EBNA2 peak position. In similar analyses, when the position of two factors (e.g., co-factors) across the genome tends to coincide, the correspondent graph shows a clear middle-point peak, as seen for the relation between EBNA2 and RBPJ (plot in the middle). Refer to the text for interpretation.

We observed that VDR binding sites are strikingly enriched for EBNA2 peaks (O/E = 96.16-fold, p < 2.0e-05;) even after controlling for DHSs track (p = 9.99e-04). EBNA2-VDR propensity to localize in similar genomic regions was confirmed by the analysis of VDR peak distribution around EBNA2 peak position (Fig. 1).

Lastly, we defined the biological meaning of genomic regions bound by EBNA2, VDR or by both using GREAT [25]. Not surprisingly, we found a strong autoimmunity signature for EBNA2-bound sites (with a *q*-value = 6.59e-07 for the ‘altered susceptibility to autoimmune disorder’ category) and several phenotypes related to immune response (e.g. B cell function, interferon gamma signaling, thymus-specific gene expression) or deregulation of pluripotency genes for the genomic intervals bound by both factors (S5 Table).

## Discussion

Looking at the distribution of EBNA2 and its co-factor RBPJ in genomic intervals associated with MS, we found that both proteins tend to fall inside MS intervals more than expected by chance. This overlap is not due to a shared preference for genomic regions of accessible chromatin, as demonstrated by the significance of the analysis (p = 9.99e-04) even after taking into account the effect of confounder track DHSs. The consistency and implications of this observation are strengthened by data showing that disease-related distributions of EBNA2 binding occur in EBV-associated autoimmune conditions (MS, SLE, RA), it is less evident in other immunological diseases not closely related to EBV (UC), and it disappears in non-immune disorders (obesity).

The biological significance of EBNA2 and RBPJ interaction with MS associated loci appears even more important if we consider that RBPJ may be related to MS pathology [29] and is a major downstream effector in NOTCH signaling pathway [30]. NOTCH-1 activity, which is also altered in MS, is up-regulated by Bacille Calmette-Guérin (BCG), whose protective effects have been reported in individuals with clinically isolated syndromes (CIS) and early MS [31, 32]. It has been proposed that BCG positive outcomes may act through the restoration of a normal NOTCH signaling [33], which can be perturbed by EBV infection via a RBPJ-mediated mechanism [34, 35]. These and other evidences implicate a role for EBNA2 and RBPJ in MS pathogenesis.

When looking at EBNA2 localization with respect to VDR genomic occupancy, we observed a striking overlap between EBNA2 and VDR binding sites. This finding was confirmed after controlling for DHSs track, thus suggesting that the tendency of EBNA2 and VDR to occupy similar genomic positions in B cell lines is not determined by a shared preference for accessible chromatin regions. Furthermore, intervals of joint EBNA2-VDR distribution significantly overlapped with MS regions more than observed for EBNA2 or VDR [14] separately. This result appears to be MS-specific when compared to results of the same analysis performed for RA and SLE.

The propensity of EBNA2 and VDR to have similar genomic localization in LCLs may be particularly relevant in the context of MS etiology. Based on the observed findings, it is possible to provide different functional interpretations for the relationship between the two proteins:

1. EBNA2 and VDR may compete for binding to MS-related sites and other genomic regions in immune cell subsets. This hypothesis is supported by some literature data showing that EBV and vitamin D tend to have antagonistic effects on B cell function: EBNA2 has selected its targets to drive B-lymphocyte proliferation and survival in primary infection [9], whereas vitamin D (and its analogs) preferentially down-regulate B cell function and play a prominent role in the maintenance of their homeostasis [36–38]. A major regulator at the crossing point between pro- and anti-apoptotic fate may be represented by *MYC*: its transactivation by EBNA2 causes continuous B-cell proliferation [9], while its suppression by 1,25(OH)2 D3 modulates cell growth [39, 40]. As far as MS is concerned, the potential antagonistic activity of these two factors in disease-associated loci may reflect the opposite tendency of EBV infection and high vitamin D levels to predispose to or protect against MS, respectively.
2. EBNA2 and VDR may interact with each other inside DNA binding sites. In this scenario, it has been shown that RBPJ is the preferential—but not exclusive—co-factor used by EBNA2 to bind to DNA, therefore a potential interaction with VDR, similarly to what has been documented for another EBV-encoded protein (EBNA3, which binds to VDR and affects its normal function) [41, 42], cannot be excluded. A further element in support of the above hypothesis is that the plot of VDR peak distribution centered on EBNA2 peak position resembles the one obtained for EBNA2 relation with its well-known co-factor RBPJ (Fig. 1). These observations may lead to speculate on two alternative outcomes for the EBNA2-VDR cooperative binding: this could either result in deactivation, as described for the EBNA3-VDR interplay [41], or in a relation analogous to that of EBNA2 with RBPJ. Once again, the latter interpretation is not supported by epidemiological evidences in MS, which suggest opposing effects of EBV and vitamin D.

One limitation of the present study is that EBNA2-RBPJ or VDR genomic distributions have been characterized by separate research groups and in different cell samples, although in both cases the analyzed lines were LCLs. Therefore, a simultaneous evaluation of the EBNA2 and VDR distribution in the same samples will be required to experimentally demonstrate whether the ability to bind to similar genomic intervals occurs in a mutually exclusive or joint (co-occupancy) fashion.

Taken together, our data suggest that EBNA2 and RBPJ tend to bind to MS-related intervals more than expected by chance; EBNA2 distribution across the genome overlaps with VDR occupancy in B cell lines, suggesting that non-heritable risk factors influence each other and interact with MS susceptibility loci in pathogenically relevant immune cell subsets [43, 44].

## Supporting Information

S1 Table EBNA2 specific binding to DNA at the base pair (bp) level (A) and analysis results for 'EBNA2 peaks' inside 'RBPJ peaks' (B). (XLS)

S2 Table EBNA2/RBPJ peak overlap with MS associated regions, defined as SNP position interval of 25, 50kb and 150 kb. (XLS)

S3 Table Shared genomic intervals between EBNA2/RPBJ peaks and MS regions (defined as SNP position +/−50kb)/MS SNPs. **A**. Shared genomic intervals between EBNA2 peaks and MS excluding MHC regions; **B**. Shared genomic intervals between EBNA2 peaks and MS regions including MHC; **C**. Shared genomic intervals between EBNA peaks and MS SNPs (original GWAS SNPs, indicated in bold, and SNPs in LD≥0.8); **D**. Shared genomic intervals between RBPJ peaks and MS excluding MHC regions; **E**. Shared genomic intervals between RBPJ peaks and MS regions including MHC; **F**. Shared genomic intervals between RBPJ peaks and MS SNPs (original GWAS SNPs, indicated in bold, and SNPs in LD≥0.8).
(XLS)

S4 Table RBPJ motif screening results for MS SNP (original GWAS SNPs + SNPs in LD≥0.8) sequences intersecting RBPJ peaks. MS SNP sequences were defined as follows: SNP position +/−100 bp. Original GWAS SNPs are indicated in bold.
(XLS)

S5 Table Prediction of function for genomic regions bound by EBNA2, VDR or by both factors. (XLS)
